# Getting time and building trust: unveiling the systemic, relational, and personal factors influencing clinical pharmacy service implementation in South Africa, a qualitative study

**DOI:** 10.1186/s12909-024-06187-3

**Published:** 2024-10-21

**Authors:** Lucille Crafford, Rashmi A Kusurkar, Elmien Bronkhorst, Andries Gous, Anouk Wouters

**Affiliations:** 1 Department of Clinical Pharmacy, School of Pharmacy, Sefako Makgatho Health Sciences University, Pretoria, 0204 South Africa; 2 Amsterdam UMC location Vrije Universiteit Amsterdam, Research in Education, De Boelelaan 1118, Amsterdam, The Netherlands; 3 LEARN! research institute for learning and education, Faculty of Psychology and Education, VU University Amsterdam, Amsterdam, The Netherlands; 4 Amsterdam Public Health, Quality of Care, Amsterdam, The Netherlands

**Keywords:** Clinical Pharmacy, Pharmacist, Interprofessional Collaboration, Motivation, Self-Determination Theory, South African Healthcare System

## Abstract

**Background:**

While multidisciplinary teams with clinical pharmacists improve medication use and outcomes, their integration in South Africa faces limitations. A lack of dedicated positions and healthcare professionals’ misunderstanding restrict ward activities and hinder full collaboration, limiting their potential to optimize patient care. This study addresses a gap by exploring how perceived healthcare professionals’ understanding of clinical pharmacists’ roles impacts their motivation and service implementation. Understanding these dynamics, particularly in resource-constrained settings, is crucial for optimizing integration and healthcare delivery.

**Methods:**

Adopting a constructivist approach, this qualitative study was conducted using focus group discussions. Through purposive sampling clinical pharmacists were recruited across South Africa’s public healthcare sector. A semi-structured guide based on previous findings explored how the perceived understanding around clinical pharmacy impacts service delivery and work motivation. Transcripts were analyzed using thematic analysis, guided by the Self-Determination Theory framework. Thematic analysis employed an inductive approach, following an initial preliminary analysis of open and selective coding to develop a coding framework.

**Results:**

Clinical pharmacists (*n* = 16) reported various challenges impacting service implementation and motivation. Two main themes were identified: (1) Time: Dedicated ward time is crucial for both the proper implementation of clinical services, as well as the clinical pharmacists’ motivation; and (2) Trust: Clinical pharmacists experience a lack of trust amongst healthcare professionals in the value of clinical pharmacy services. The themes illustrated mechanisms at work at three levels: systemic (lack of dedicated positions), inter-relational (healthcare professional’s misconceptions), and personal (thwarted autonomy).

**Conclusions:**

Systemic challenges, like the absence of official positions present the biggest obstacle, affecting support, scope of practice, and healthcare professional interactions. While systemic changes are crucial for full integration of clinical pharmacists, in resource-constrained settings fostering autonomous motivation is equally important. This study emphasizes the need for a multi-faceted approach, including policy changes, international collaboration, interprofessional education, and interventions to empower clinical pharmacists for proactive service delivery. By addressing these interconnected challenges, healthcare systems can leverage the full potential of clinical pharmacists, ultimately improving healthcare delivery and patient outcomes.

**Supplementary Information:**

The online version contains supplementary material available at 10.1186/s12909-024-06187-3.

## Background

 Including clinical pharmacists in multidisciplinary teams and supporting their role in clinical decision-making are well-established methods for improving medication use, continuity of care, and patient outcomes [1–5]. Globally, full-time clinical pharmacists bridge healthcare gaps related to the optimal use of medicines, improving patient outcomes within teams^6– 9^. This aligns with South Africa’s experiences in services like medication reconciliation [10], anticoagulation services [11] and antibiotic stewardship [12]. However, despite ongoing efforts, South Africa currently lacks formalized competency standards for clinical pharmacists. While these standards are in development [13], specialist roles remain less established compared to some European countries, with no specific requirements and performance indicators (14, 15). This absence poses challenges in assessing the quality and consistency of clinical pharmacy services, raising concerns about the effectiveness of the practice without established benchmarks. Recognizing context- specific challenges and adapting best practices from other countries could help South Africa fully leverage the potential of clinical pharmacists.

Clinical pharmacy services in South Africa began developing in the 1980s, driven by efforts to expand the pharmacist’s role beyond dispensing [16]. Despite initial resistance, clinical pharmacy evolved toward patient-centered care, with increasing focus on comprehensive medication management [14, 17]. The establishment of the South African Society of Clinical Pharmacy (SASOCP) in 2011 further promoted the integration of clinical pharmacists into healthcare teams and the rational use of medicines [18].

South Africa’s two-tiered healthcare system is marked with significant inequalities. While 81% of healthcare spending occurs in the private sector, it serves only about 27% of the population. The underfunded public sector, serving the majority, struggles with limited resources to activate and maintain clinical pharmacist posts, despite their recognized need in ward-based care [19–22]. As of 2015, there were only 0.629 pharmacists per 1,000 people, with most working in the private sector, further exacerbating shortages in public healthcare [23]. The National Health Insurance (NHI) system, introduced by the Department of Health in 2011 to integrate care across sectors, faces financial and operational challenges, creating uncertainty about its viability [21]. These issues continue to hinder, and likely exacerbate, the full integration of clinical pharmacists into multidisciplinary teams, especially in rural and under-resourced areas [22].

Clinical pharmacists have increasingly adopted collaborative and bedside care models [5, 24–26], leveraging their expertise to bridge medication-management gaps and improve quality of care [27]. This aligns with the evolving hospital pharmacy landscape where pharmacists’ medication expertise is crucial for complex cases and seamless care transitions, presenting opportunities to deliver patient-centered care within collaborative team settings [28]. This trend coincides with the global healthcare landscape’s shortage of qualified professionals, highlighting the need to optimize the roles of existing personnel such as clinical pharmacists [29].

**Fig. 1:**
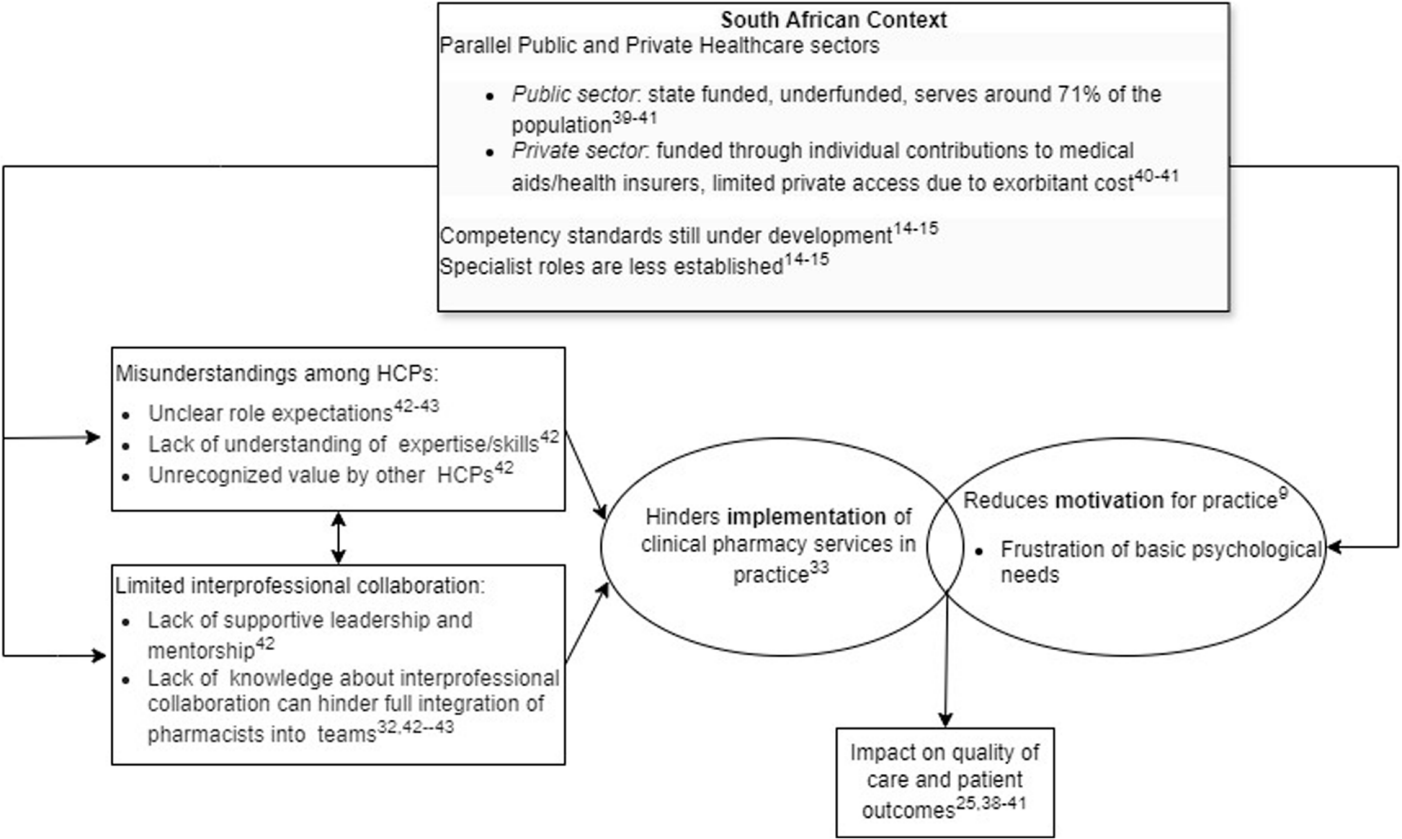
Interplay of contextual factors, service implementation, pharmacist motivation, and patient outcomes

However, effective integration requires developing strong collaborative relationships [30–33]. A major barrier to collaboration and service implementation is the misunderstanding among healthcare professionals (HCPs) regarding the role and competencies of clinical pharmacists [32, 34–36]. This lack of clarity can be further exacerbated by a lack of autonomy, as highlighted by The Self-Determination Theory (SDT). Within this framework, a sense of autonomy consistently predicts team effectiveness, dedication, and goal accomplishment (37, 38). This limited understanding can be attributed to several factors specific to the South African healthcare context. The country faces a complex healthcare landscape with a dual public and private system, resource constraints, and a shortage of qualified personnel (39, 40). Additionally, while competency standards are being developed for clinical pharmacists [13], specialist roles remain less established compared to some European countries, lacking specific requirements as well as performance indicators [15]. This lack of clarity around roles can contribute to HCP’s misunderstanding about them. Figure 1 illustrates the interplay between contextual factors, service implementation, pharmacist motivation, and patient outcomes.

This limited understanding and underutilization of clinical pharmacists’ roles can negatively impact their motivation, potentially leading to feelings of frustration and reduced engagement with their roles. The Self-Determination Theory emphasizes the quality of motivation, differentiating between autonomous motivation (arising from personal interest) and controlled motivation (driven by external pressures) [49]. Fulfilling basic psychological needs of autonomy (self-directedness), competence (capability), and relatedness (connection) is essential for autonomous motivation, which is linked to well-being, better healthcare professional performance, and improved service implementation [49–51]. Conversely, frustration of these needs can lead to controlled motivation, associated with burnout [49, 52]. Members of high-performing collaborative teams value interprofessional collaboration and demonstrate autonomous motivation, which is linked to positive behavioral engagement and improved team outcomes (37, 38). However, research exploring the impact of HCP motivation on successful multidisciplinary teams and service delivery remains limited (37, 38). In our previous South African study [43] we found high scores for both autonomous and controlled motivation among clinical pharmacists. Support from colleagues was found to foster the preferred autonomous motivation, leading to improved well-being and service delivery [43].

Thus, research suggests a link between inaccurate perceptions held by HCPs regarding clinical pharmacists’ roles and lower levels of confidence among clinical pharmacists in their practice. In an earlier quantitative study, we investigated the understanding of HCPs towards the roles and competencies of clinical pharmacists in South Africa [41]. This research highlighted the potential effect of unclear role expectations and a lack of understanding amongst HCPs, leading to reduced utilization of pharmacists’ expertise and hindered team collaboration towards patient care. Using the theoretical lens of SDT, the current study aims to provide: (1) a deeper understanding of how clinical pharmacists perceive the understanding of other HCPs of their role, and (2) how this perception impacts actual clinical service implementation and the motivation of clinical pharmacists.

### Research questions

- How do clinical pharmacists perceive the understanding that other healthcare professionals have of their role, in delivering clinical services?
- How does the perceived understanding of HCPs of the role of a clinical pharmacist affect the implementation of clinical services (profession of clinical pharmacy), and the motivation of clinical pharmacists?

## Methodology

### Study design

We employed a qualitative research design, guided by a constructivist paradigm, to explore clinical pharmacists’ experiences. Focus group discussions were the primary method of data collection. To address recruitment challenges, we adapted our approach, employing both focus group discussions and interviews, as both techniques allowed for in-depth discussions (53, 54). Throughout data collection and analysis, we considered both individual experiences and the dynamics of researcher-participant interactions [55]. For this study, “clinical pharmacist” refers to individuals with a Master of Pharmacy (M.Pharm) degree in Clinical Pharmacy. This study adheres to the COREQ guidelines for reporting qualitative research [55].

### Study setting and population

Using purposive sampling, we contacted all members of the South African Society of Clinical Pharmacists (SASOCP), known to be practicing in hospitals with clinical pharmacy services. Nineteen pharmacists met the inclusion criteria (holding a clinical pharmacy qualification and actively involved in service delivery), of which 16 consented to participate and were subsequently contacted to schedule interviews (telephone/email).

By reaching out to the majority of clinical pharmacists working in the public sector our approach ensured the inclusion of a range of experiences, including those who were actively involved in direct patient care, as well as those who balanced clinical and dispensing duties, providing insights into the varied roles within the profession. The participants also varied in their years of clinical experience, ranging from newly qualified pharmacists with 1–2 years of practice to more experienced pharmacists with over 10 years of clinical work. Our final sample represented both urban and rural public healthcare institutions. It represents a significant portion of clinical pharmacists in South Africa’s public sector, enhancing the study’s representativeness. Currently, in South Africa, no official job descriptions for clinical pharmacists exist, as they are not able to register as specialists at the South African Pharmacy Council [18].

### Data collection

In 2022, four focus groups were held: two online (Zoom) and two in-person (in a separate room located at the university). All participants granted audio recording permission. Informed by our previous quantitative study [41], the focus group guide (Appendix A) explored how HCPs’ perceptions/understanding of clinical pharmacy influence service implementation and clinical pharmacist work motivation. We investigated clinical pharmacists’ experiences with current interprofessional collaborative practices and the impact of HCPs’ understanding on their inclusion in multidisciplinary teams. Questions were designed to capture experiences related to autonomy, competence and relatedness. For example, exploring their sense of competence when providing clinical services and the quality of their interactions with other HCPs, reflecting the need for relatedness. Background data (age, gender, experience) were collected. All interviews were conducted and transcribed verbatim in English. No follow-up interviews were carried out.

### Data analysis

Thematic analysis (open and selective coding) was employed using ATLAS.ti [56]. First author (LC) conducted line-by-line coding of all interviews to generate initial descriptive codes. To ensure coding trustworthiness, AW independently coded a subset of transcripts. Participant validation of the transcripts was not performed. Instead, we used alternative methods such as independent coding, and iterative analysis to ensure the credibility of our findings [57]. An inductive approach was then used to organize these codes into sub-themes, forming the basis of a preliminary coding framework that evolved as new insights were developed. Iterative discussions between LC and AW facilitated consensus, ensuring that the coding scheme remained responsive to participants’ experiences and enabled the grouping of similar codes into broader categories. For example, codes related to “lack of professional recognition,” “collaborative practice challenges,” and “role ambiguity” were grouped into larger themes reflecting the clinical pharmacists’ experiences. These themes were then refined through the lens of SDT, focusing on how participants’ experiences related to the basic psychological needs of autonomy (control over clinical activities), competence (confidence and skill levels), and relatedness (interactions with other healthcare professionals). This SDT-guided approach allowed us to identify how misunderstandings about clinical pharmacists’ roles impacted their intrinsic motivation and the implementation of clinical services. Data sufficiency was considered when no new codes were identified, ensuring rigor in both the analytical process (analytical sufficiency) and the richness of the data generated [58].

### Ethical considerations

Ethical approval was obtained from Sefako Makgatho Health Sciences University (SMUREC/P/232/2020:PG). Informed consent included explaining voluntariness, confidentiality, and anonymity. No incentives were offered.

### Reflexivity

The research team comprised three clinical pharmacists, a psychologist, and a medical doctor offering diverse perspectives in medical education, qualitative research, and clinical pharmacy. LC, the interviewing pharmacist, also teaches in the M.Pharm programme. This position enabled interpreting the data and events described by the participants. As the interviews were relating to experiences in practice and not related to the education received, this presumably has not influenced data collection. While aware of potential researcher influence during interviews, LC maintained an open mind to avoid biasing data collection and to use her own experiences merely as a lens to obtain understanding of participants’ experiences. All codes were discussed with a team member, and final themes were confirmed by the entire team. LC completed a Qualitative Data Analysis Course, and RK, EB, AG, and AW are experienced researchers in qualitative studies and have collectively published numerous research articles. RK, AW and LC also have expertise in motivation research, particularly in SDT.

## Results

Sixteen pharmacists (employed 9–20 years, delivering clinical services 1–11 years) participated in three focus groups (2–4 participants, 50–70 min each). One additional individual interview was conducted with a participant who could not join the focus groups, to ensure all perspectives were included. Of the participants, eight were from urban healthcare settings and eight from rural areas. On average, participants were seven years post-graduation and dedicated five hours daily to clinical services. Notably, only five had full-time opportunities for ward-based services, where their employer provided them with dedicated time, while the others balanced clinical and dispensing duties. Most participants were actively involved in direct patient care, though a few had more limited involvement due to their roles combining both clinical and non-clinical responsibilities.

As participants described their experiences and perceptions regarding HCPs understanding of their role and how this affects service implementation and their work motivation, various other challenges were described. Two main themes were developed and relate to (1) Time: Dedicated ward time is crucial for both the proper implementation of clinical services, as well as the clinical pharmacists’ motivation; and (2) Trust: Clinical pharmacists experience a lack of trust amongst HCPs in the value of clinical pharmacy services.

Quotes illustrating Theme 1 and 2 are provided in Table 1.

**Table 1:** Participant quotes supporting Theme 1 and Theme 2:

**Theme 1 on how dedicated ward time is crucial for both the implementation of clinical services, as well as motivation**		
**Quote**	**Quote # in text**	**Participant #**
***Implementation***		
“Personally I think I am ready to practice, it’s just unfortunate that I don’t have enough time, but I will create time. We are given the skill when we are studying, we have the knowledge. I just need time and then that’s it.”	1	5
“I feel I’m prepared. It’s just that I need a bit more support from the pharmacy management. Because we already have support from the HOD’s [Head of Departments] and the hospital. But not pharmacy management itself, we need support from them to provide clinical pharmacy service in this hospital.”	2	10
***Relatedness***		
“I have a lot of good things to say with regards to these relationships that I have built with them. I think with time, as well as proximity- for you to be available when they need you and to be visible most of the time - this really helps create that positive perception and attitude of clinical pharmacists from their side. They will show that they really appreciate what we are doing in terms of rendering clinical service.”	3	4
“Motivation comes from the doctors that are actually working in a team with you. When you go with the medical team and they don’t give that much support, I always get drained. But I always get excited when I go to the surgical section, because they recognize me. So, recognition also builds up to motivation.”	4	5
“I am all by myself, clinical pharmacy is not established at all, so I don’t have anyone helping me, which is really lonely, and I think I could be doing much more if I had like a support system or a mentor or somebody to guide me. But that’s a really sad thing actually. And then, cause like I wanted to do therapeutic drug monitoring, but I am just not comfortable doing it by myself. I need somebody to do it with me, but there isn’t anyone. Nobody does it, nobody knows how to do it. Coming from the private sector, there was a whole clinical pharmacy programme and support system. Now I am by myself, so if I want to expand or do something else, it’s up to me to teach myself basically. But then if we had more staff then I could expand into the rest of the hospital, or not be pulled back to the pharmacy as much that would be superb.”	5	12
“I got a lot of support from the paediatric department, from the head of paediatrics, the doctors and everything, so that motivates me as well. But it would be nice if there is a post for it, If the post is there you don’t have to worry.”	6	2
***Competence***		
“I could be more ready. I can’t say I feel 100% ready for practice, but I think the only way really to be ready is to start doing the work, to practice it consistently…being in the wards full-time, will help us to actually do it. The ones in the wards will be able to see all that needs to be seen, learn all the tricks and gain experience.”	7	4
“Knowing that you are making a difference in people’s lives, and also you feel different- knowing you have the clinical theoretical knowledge and now you are able to be a different kind of pharmacist”	8	11
“In addition to guidelines and published articles, what also really helps me is teaching students, because when you teach you actually reinforce what you know and it becomes easy to relate what you have been teaching to some of your students, or to a team of doctors. You gain confidence, because when you teach you have been saying things so many times. I have also realized that in the main pharmacy, in general we don’t say much, we just do what we do, but we never explain why we do what we do. So I think that skill is not really developed, but when you are with students, you learn to really express your thoughts and that then helps you when you are engaging with the doctors. I think teaching, in itself, is very important”	9	16
***Autonomy***		
“Pharmacy management sometimes resists ward rounds due to concerns that the number of patient medications we dispense will decrease. Their focus on meeting targets clashes with the need for clinical pharmacy. Our ward-related outcomes are not even included in the pharmacy meetings, I have to go around people, asking the pharmacy manager for support.”	10	7
“It’s more the manager who sees that the pharmacy is not coping and then pulls you back to help the team.”	11	1
“There is no Standard Operating Procedures in the hospital, there is no clear goal of what we need to do. That’s one of the barriers and leading to others also not knowing what a clinical pharmacist does. So, it’s solely on you if you go and study clinical pharmacy, to come back and make sure that it’s done, but there’s a lack of structure...that’s the reason for the slow pace towards implementing clinical pharmacy, especially in our institution.”	12	11
“At first when we get to the wards, they will see you as a person that is there to actually manage their stock, they will tell you that they are out of stock, or whatever items they need in the pharmacy. Sometimes you want to say no, but you just say yes: I will do it. But I think as time goes by, they get used to seeing you doing other things, and that’s when they start understanding. But at first, you are just there for stock control.”	13	2
“I think we are the middle man, or the middle person in between whatever the pharmacy is offering and the nurses actually require. So, I think there was a bit of a gap between us. Maybe the gap was created by the pharmacy counter, but the minute they saw us there, they actually see that they need us, or they actually indicate as well: we need you guys to be here. I think we are more accessible when we’re in the wards than when we are behind the counter.”	14	16
“I don't know why pharmacy colleagues resist. Maybe they oppose our progress or prioritize outpatients. Their initial resistance has not yet fully subsided, although we are trying our best to make sure that they understand what we are doing. They don't seem to appreciate our work despite efforts and positive results. I'm unsure of the reason.”	15	11
“The worst hostility comes from pharmacy staff. It's disheartening because our hospital has the most trained pharmacists. We should be leading clinical pharmacy, yet we lack internal support. We wonder if it's lack of knowledge or resistance to the new role. We face comments like ‘you want to be a doctor all of a sudden, are you not one of us ' and ‘do you think you’re better than us’?”	16	2
**Theme 2 on how clinical pharmacists experience a lack of trust amongst healthcare professionals in the value of their clinical pharmacy services**		
**Quote**	**Quote # in text**	**Participant #**
**Pressure to justify work**		
“With the consideration of allocating a clinical pharmacist to the wards, there were a lot of meetings where people were asking questions as to why is it that these guys have to go to the wards full time. There were a lot of questions to the fact that there has got to be a report that should come from them and that their work should be measured. But the question that I am asking myself is: why is it that these guys who are going to the wards, who are rendering clinical activities and has more knowledge, why is it that they should be measured and the guys who are in the main pharmacy are not measured, or their work is not measured. If there is no support for clinical pharmacy, it really puts a strain on us. We may implement it, but it puts a lot of strain, because it is like you must look behind your back and see whether there is nobody going to fight, and maybe say: what these guys are doing is purely a waste of time”	17	1
“They want to see exactly what has been done by the clinical pharmacists, how many interventions have been made, also maybe how much money have we saved. I mean we sat for hours in the boardroom, where they said we should be stating one by one what is it specifically that the clinical pharmacists must be able to show when they come back from the wards, or according to them (other pharmacists) it is useless - if they are not able to provide this, maybe they should rethink it.”	18	4
“When I go and check on the patients, maybe I am reading up on something and they will come around and say: ‘What did we do wrong now?’ Sometimes they feel like I am an investigator spying on their work, an intruder.”	19	6
“Initially when I started there was a lot of negativity from some of the doctors. I remember introducing myself to one of the consultants, and he actually corrected my grammar. So at first it was like a war. I think they didn’t know what we wanted to do in the wards. It took time, discussions and meetings to actually come to an agreement that we will attend the ward rounds, because when we first introduced ourselves, everyone was like: ‘What you be there in the wards for?’. It took time for them to understand and see our contribution in the grand rounds. However, over time they understand you better, especially when they see the results of what you are doing, but initially they didn’t accommodate us.”	20	4
“We are at the point where doctors and nurses are actually more ready for clinical pharmacy services and they are ready to work as a team. Due to the fact that there has been a more permanent clinical pharmacist, they have been able to see the need for us, see the care we provide, and they are now realizing what we can offer.”	21	9
“I do like a really comprehensive report of about 20 pages with each intervention that I make and meetings I attend, so at least the manager can see what I am doing. I also do stats on all the patients I see on a daily basis, and I graph it. It’s all there for her to see. Thankfully for me, she gets good feedback from the rest of the doctors. I am also on a few committees, like the Antimicrobial Stewardship committee, and as long as all those committees function she has never had an issue with my work. It’s just that when the pharmacy is short staffed, then I am still the first person to get called back. The report is my own initiative, so they can see when I am not around in the pharmacy, I am actually doing the work. The saying goes, ‘if you don’t document it, it’s not done’. And unfortunately I feel I need some record of what I do.”	22	12
**Impact on Integration and Collaboration**		
“This morning I was speaking to a family physician, and he said he started a mental health multidisciplinary team with meetings on Mondays and then he mentioned everyone else and didn’t mention pharmacy, and I was disturbed the whole day. What I am trying to say is that: we need to push ourselves, make ourselves seen, spend consistent time in the wards and document what we do. Because then when you give them the report that shows your role and why you need to be there. They can’t deny that.”	23	11
**Resistance from Pharmacy Colleagues**		
“Sometimes when they are overwhelmed, then they see whatever you are doing outside of the pharmacy not as important, the main thing is to just push the queues. For them, whether they understand our role or not, is not important…sometimes it’s not that they don’t understand the role, they understand it- it’s just that when they get overwhelmed, then the role is not as important to them.”	24	2
“Only where there are enough pharmacists that are allocated to do the things in the pharmacy, you don’t need to actually take from another section to cover the dispensary. So where there is enough staff in the dispensary, or there is somebody hired specifically for clinical pharmacy services, it might bring some space for moving around, or even the hospital and pharmacy managers supporting us.”	25	6

### Theme 1

Time: Dedicated ward time is crucial for both the proper implementation of clinical services, as well as the clinical pharmacists’ motivation.

This section explores how implementation of clinical services is impacted by dedicated ward time. It also describes how ward time affects clinical pharmacists’ motivation through fulfillment and/or frustration of their needs for relatedness, competence and autonomy.

#### Implementation

Participants expressed their confidence to practice, but alluded to the lack of available time and how it is linked to support received from management (Quote 1; Q1, Q2).

#### Relatedness

Increased ward presence fostered positive interactions with doctors and a sense of belonging (Q3). Recognition for their contributions further motivated pharmacists, as highlighted by quote 4. On the other hand, feeling undervalued by other HCPs, often due to role misunderstanding, also contributed to a lack of relatedness.

Participants felt isolated due to a lack of support networks, including mentors or other clinical pharmacists (Q5). This limited their confidence and service delivery. They desired a support system or mentor but felt alone and unable to expand their practice without guidance (Q5).

Even when they did receive support, participants emphasized the need for official clinical pharmacist positions (Q6). This would eliminate the need for individual advocacy and ensure service continuity (Q6).

#### Competence

Ward experience was seen as crucial for building confidence for clinical practice. Some participants desired more time for service delivery (Q7). Furthermore, perceived competence and witnessing the impact of their work boosted motivation. Participants felt motivated knowing they made a difference (Q8) and by activities that reinforced their skills (Q9).

#### Autonomy

Clinical pharmacists perceived limited autonomy with regards to their time spent, which further hindered service delivery. Pharmacy management’s focus on dispensing metrics (Q10, Q11) created a conflict, as pharmacists needed additional support to dedicate time to clinical duties in the wards. This lack of control over workload allocation often resulted in them being pulled back to the dispensary when staffing shortages occurred (Q11). Furthermore, the absence of standardized procedures for clinical pharmacists (Q12) contributed to misunderstandings and resistance among other HCPs. This lack of clarity around their role and responsibilities (Q12) likely played a part in how they were initially perceived by nurses, who primarily saw them as stock controllers (Q13). While these perceptions improved over time as other HCPs witnessed the value of their contributions (Q13, Q14), the most significant resistance came from within pharmacy itself (Q15, Q16). Participants reported needing to justify their work to pharmacy colleagues (Q15, Q16), suggesting a lack of recognition and acceptance of their expanded role.

### Theme 2

Trust: Clinical pharmacists experience a lack of trust amongst healthcare professionals in the value of clinical pharmacy services.

This theme covers how clinical pharmacists experience a lack of trust in them and their role in the hospital setting. This manifests in ways that hinder their ability to integrate and contribute their expertise.

#### Pressure to justify work

Due to the lack of established positions and a clear scope of practice, participants felt compelled to extensively document their ward activities (Q17, Q18). They described pressure to quantify their contributions and demonstrate their value to other HCPs. This perceived lack of trust was further amplified by limited exposure, as clinical pharmacists felt that some doctors and nurses initially held negative attitudes due to a lack of familiarity with clinical pharmacy (Q19, Q20). However, pharmacists working alongside other HCPs on the wards fostered better understanding and collaboration (Q21), highlighting the importance of ongoing interaction to bridge this gap. While some clinical pharmacists documented activities comprehensively, they still faced being pulled back to dispensing duties when the pharmacy was short-staffed (Q22). This, along with the reporting obligation, raised concerns about micromanagement and a lack of trust in their decision-making, further hindering their autonomy (Q22).

#### Impact on integration and collaboration

Participants described feeling a need to prove themselves to other HCPs for integration in teams (Q23). This stemmed from the lack of official clinical pharmacist positions, hindering their ability to be consistently present in wards and display their contributions (Q23).

They mentioned justifying their role and documenting activities due to this unclear role (Q23).

#### Resistance from pharmacy colleagues

Many clinical pharmacists reported experiencing resistance from their pharmacy colleagues when attempting to integrate into ward teams. This resistance manifested in several ways. Potential reasons behind this resistance, based on the participants’ perspectives included uncertainty about the evolving role of clinical pharmacists or competition anxieties among pharmacists (Q24). Additionally, clinical pharmacists reported feeling pressure from colleagues overwhelmed by their own workload, who perceived ward activities as less important (Q24). This highlights a domino effect: the lack of established clinical pharmacist positions and resources creates challenges for both clinical pharmacists and their colleagues, ultimately impacting integration within the team.

Limited ward time, due to staffing pressures in dispensing, and the need to justify ward activities exemplify the strain on existing staff and the potential lack of trust in their expertise (Q25). This participant highlights that if there were enough pharmacists specifically allocated to clinical services, it would free up time for ward activities and potentially reduce the pressure to justify their work.

**Fig. 2:**
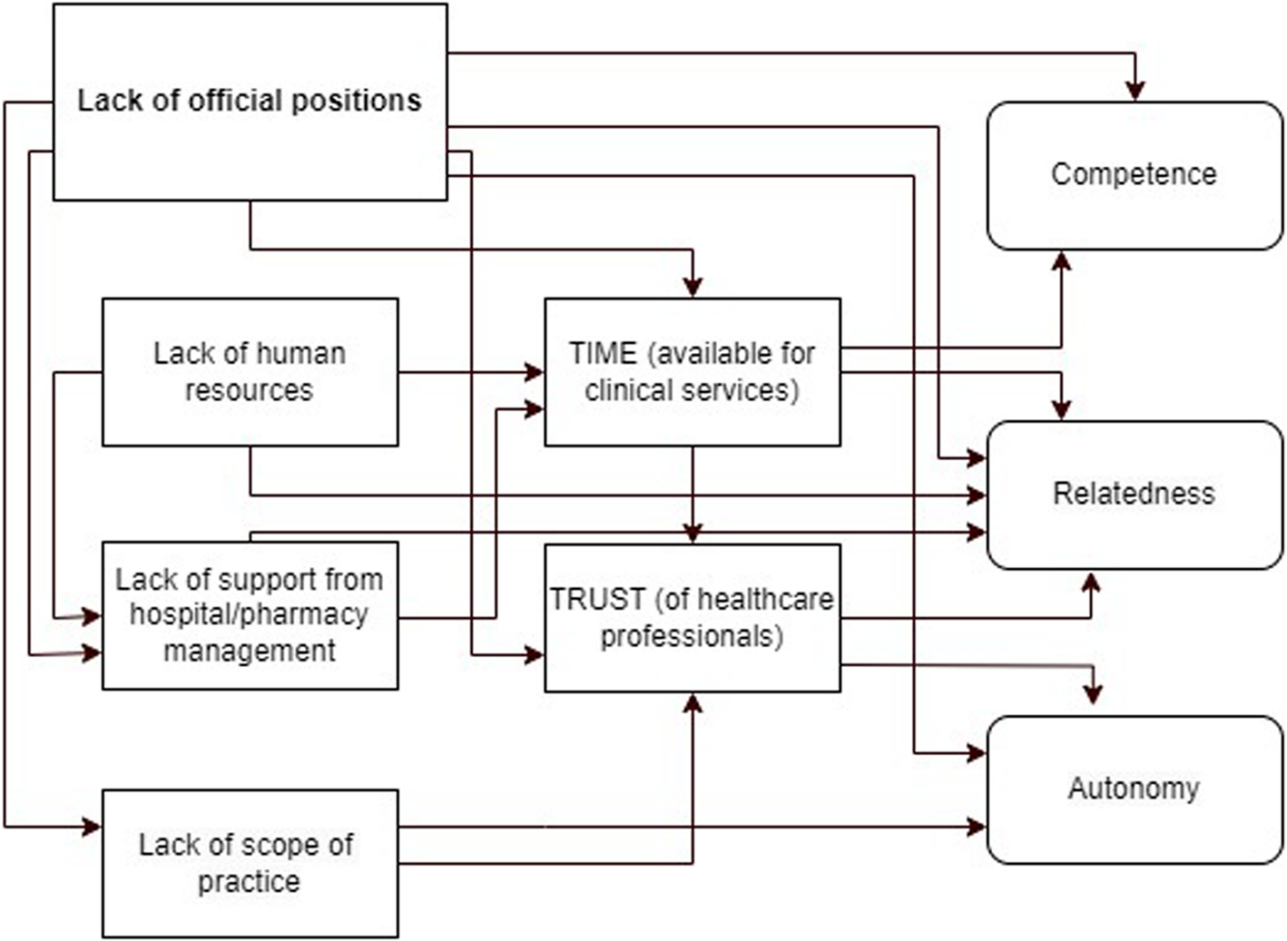
Flowchart: Impact of Challenging Factors on Clinical Pharmacist Motivation and Service Implementation

Figure 2 visually represents the interconnectedness of the challenging factors identified in the interviews. It demonstrates how these factors impact the time available for delivering clinical services, trust among HCPs (due to misconceptions), and ultimately, the basic psychological needs and work motivation of the clinical pharmacists. The lack of official clinical pharmacist positions emerged as a central theme impacting various interconnected aspects of their role, ultimately affecting service implementation and work motivation (see Fig. 2). These findings highlight a system lacking trust in the value of clinical pharmacy services. The extensive documentation requirements, limitations on autonomy, and resistance from colleagues create significant barriers for CPs to integrate effectively and contribute their expertise.

## Discussion

This study explored how clinical pharmacists perceive their role to be viewed by other HCPs, and how this affects their motivation and service implementation in South Africa’s public healthcare sector. Findings reveal a perceived lack of trust in the value of clinical pharmacy services, primarily stemming from the lack of established clinical pharmacist positions. This lack of positions directly impacts dedicated ward time, hindering their ability to effectively deliver services and build trust with other HCPs.

Our findings confirm previously identified challenges, including lack of formal recognition, funding, human resources, and understanding among other HCPs [14, 18, 41, 43]. They add to the literature by demonstrating how these challenges interlink and identify the most impactful ones for prioritizing interventions and achieving change. The absence of formal clinical pharmacist positions limits dedicated resources and support for ward-based activities, consequently hindering both relationship building with other HCPs due to misunderstanding and resistance, as well as opportunities to demonstrate the value of their services. While other HCPS may be initially uncertain, they tend to become more receptive and appreciative of clinical pharmacists’ roles over time. However, our findings suggest that a significant portion of the resistance stems from pharmacists themselves, possibly due to role ambiguity, perceived competition, or feeling overwhelmed — all of which are exacerbated by systemic challenges. This internal resistance, alongside external misunderstandings complicate efforts to fully integrate clinical pharmacy services into multidisciplinary teams, as identified in our earlier research [41]. Furthermore, by frustrating the basic psychological needs of clinical pharmacists (lack of autonomy as they have to prove their worth; lack of competence as confidence develops with time; and lack of relatedness due to strained relationships with HCPs), the system undermines their autonomous motivation. Ultimately, this misunderstanding and resistance among other HCPs, compounded by limited ward time and unclear roles, can hinder effective collaboration and compromise the full potential of clinical pharmacy services. This could lead to decreased job satisfaction [43, 59], controlled motivation [60], and potential workforce attrition (61, 62), jeopardizing the future of clinical pharmacy services. Our research sheds light on how systemic, inter-relational, and personal factors impact clinical pharmacists’ basic psychological needs, offering valuable insights into their needs and challenges within an already burdened public healthcare system.

As previously reported [43], the lack of dedicated positions in South Africa thwarts clinical pharmacists’ autonomy and relatedness, leading to need frustration. Aligning with SDT (63, 64), our study further exemplifies this, demonstrating how limitations on task choice restrict autonomy, competence is undermined when one is told one cannot experience effectance in action, and sense of belonging is threatened by exclusion/rejection. Research [65] highlighted that actively thwarted needs, as opposed to simply unmet ones, hold a stronger influence - frustration predicts negative outcomes even exceeding the influence of need satisfaction (66, 67). This underscores the importance of addressing these needs and fostering work environments that prevent frustration, optimizing well-being, motivation, and performance [52, 68]. Our research reveals the interplay of organizational challenges, interpersonal dynamics, and individual needs (thwarted basic psychological needs) in hindering clinical pharmacist integration and individual motivation.

This study reinforces the need for clear expectations and standardized guidelines for clinical pharmacy, as advocated by previous research (14, 15, 45). The absence of clear guidelines creates ambiguity and colleagues could perceive undefined roles as a threat [32, 41]. Building on this, our findings demonstrate this resistance, stemming from colleagues’ limited understanding of clinical pharmacists’ scope and perceived benefits. Participants reported misperceptions of clinical pharmacists “inventing” a role for personal gain or avoiding responsibilities. This underscores the need for improved communication and education to showcase their value and promote collaboration.

Our findings extend beyond South Africa, resonating with resource-limited regions (Africa, Latin America, Middle East, parts of Asia and Europe) where ward-based pharmacy services are in development. While context is crucial, understanding South Africa’s experiences can inform service development in comparable settings. Successful adaptation in each unique setting requires considering local regulations, resource constraints, and professional dynamics. Standardized guidelines, across public and private sectors, would define practice scope, expectations, and support the National Department of Health’s vision (69, 70), addressing resistance within pharmacy teams. This aligns with our prior call for integrated services [41], both calling for a shift away from pharmacists “proving themselves” due to the lack of official positions [34] toward a novel multidisciplinary model where clinical pharmacists participate in decision-making. Limited ward time, as identified previously [41], hinders interaction with other HCPs and fuels misunderstanding and resistance. Our study reaffirms the importance of fostering multidisciplinary interactions and improving HCP knowledge about clinical pharmacy, aligning with prior research (5, 31, 32, 41, 71). Enhanced understanding is linked to greater acceptance and improved healthcare quality [5, 32, 41]. Ultimately, our research underscores the need for system-level changes to fully integrate and leverage the expertise of clinical pharmacists.

### Limitations

The sample size was relatively small. However, this study was conducted in the context of South Africa, where ward-based clinical pharmacists remain scarce. Our sample therefore effectively reflects the demographics of existing practitioners in public institutions. The experiences and challenges faced by pharmacists in the private sector may differ, requiring further research. Recognizing the limitations of direct applicability to other healthcare contexts, the findings remain relevant for regions with similar challenges.

### Implications for clinical practice/health policy

Most salient is the need for systemic reforms for full integration of clinical pharmacists. Despite two decades of training and education, established clinical pharmacy services remain elusive in South Africa. A multifaceted approach is needed [72]: “top-down” changes, including national policies supporting dedicated positions, legislative frameworks for full practice scope, and reimbursement for patient-centered services. Additionally, to enhance the credibility of clinical pharmacy services, a collaborative effort could be undertaken to develop a “bottom-up” accreditation process. This could potentially be initiated by the South African Society of Clinical Pharmacy, establishing standards and benchmarks for service delivery. Collaborations with other national and international societies could further assist to legitimize the accreditation and pave the way for national recognition. Learning from countries that have successfully integrated clinical pharmacists and adapting it to the local context can unlock the potential of these specialists, improving medication use, patient outcomes, and healthcare system efficiency.

Short term goals include fostering intrinsic motivation through need satisfaction, which will provide a valuable tool for navigating the current landscape and empowering pharmacists to be resilient and innovative contributors to healthcare delivery. In resource-limited settings like South Africa, fostering self-determined motivation (driven by need satisfaction) is crucial for clinical pharmacists navigating uncertainty and interdependence. Future research examining how basic psychological needs: satisfaction or frustration, e.g., related to lack of autonomy, influence pharmacists’ adaptability and proactive performance in these challenging environments is recommended. Adaptive (coping with and responding to change) and proactive (initiating change) performance is known to be promoted by satisfying the basic psychological needs (73, 74). Such research could inform interventions empowering pharmacists to leverage uncertainty for improved patient care, even without ideal systemic support.

## Conclusion

By unlocking their full potential, clinical pharmacists can improve medication use, patient outcomes, and healthcare efficiency. This study identified systemic, inter-relational, and personal challenges such as hindering clinical pharmacist integration in South Africa’s public healthcare. To address these interconnected challenges, a multi-faceted approach is recommended. This includes national policy changes, collaboration for accreditation, interprofessional education, and interventions to empower clinical pharmacists for proactive service delivery.

## Supplementary Information

Supplementary Material 1.

## Data Availability

Anonymized datasets used and analyzed are available from the corresponding author on request.

## Abbreviations

HCP: Healthcare Professional
SASOCP: South African Society of Clinical Pharmacy
NHI: National Health Insurance
SDT: Self-Determination Theory
COREQ: The Consolidated Criteria for Reporting Qualitative Research
M.Pharm: Master of Pharmacy
