# Supplementary material for: Getting time and building trust: unveiling the systemic, relational, and personal factors influencing clinical pharmacy service implementation in South Africa, a qualitative study
Source: BMC Med Educ. 2024 Oct 21;24:1179. doi: 10.1186/s12909-024-06187-3 (PMC11492519; doi:10.1186/s12909-024-06187-3)
Supplement: Supplementary file 1 — Supplementary Material 1. [file 12909_2024_6187_MOESM1_ESM.docx]

**Focus Group Discussion Guide**

1. How do you experience the attitude of nurses toward the services you deliver in the ward?
2. How do you experience the attitude of doctors toward the services you deliver in the ward?
3. Do you feel that the doctors understand your role as a clinical pharmacist in the ward? Why do you think they do/don’t understand your role?
4. Do you feel that the nurses understand your role as a clinical pharmacist in the ward? Why do you think they do/don’t understand your role?
5. Do you feel that the perception or understanding of other healthcare professionals on your role has an influence on your motivation to deliver clinical pharmacy related services? Why and how?
6. How does this translate to how you work together with the other healthcare professionals?
7. How does this make you feel about your own practice, as clinical pharmacist?
8. How do you experience the attitude of other pharmacists, as well as the pharmacy management when leaving the pharmacy to perform ward based functions?
9. Do you feel that the other pharmacists understand your role as a clinical pharmacist in the ward? Why do you think they do/don’t understand your role?
10. Do you feel that the understanding of other pharmacists on your role has an influence on your motivation to deliver clinical pharmacy related services? Why and how?
11. Do you feel supported by other healthcare professionals? How do they respond to you being a clinical pharmacist?
12. Do you feel motivated to leave the pharmacy to deliver ward-based clinical services? Why (not)?
13. (How) do you cope with the mentioned barriers that demotivate you?
14. What do you need to feel supported and prepared/ready to deliver clinical services?
15. Would you like to give additional comments?
